# Control of Fingertip Forces in Young and Older Adults Pressing against Fixed Low- and High-Friction Surfaces

**DOI:** 10.1371/journal.pone.0048193

**Published:** 2012-10-24

**Authors:** Kevin G. Keenan, William V. Massey

**Affiliations:** Department of Kinesiology, University of Wisconsin-Milwaukee, Milwaukee, Wisconsin, United States of America; Universidad Europea de Madrid, Spain

## Abstract

Mobile computing devices (e.g., smartphones and tablets) that have low-friction surfaces require well-directed fingertip forces of sufficient and precise magnitudes for proper use. Although general impairments in manual dexterity are well-documented in older adults, it is unclear how these sensorimotor impairments influence the ability of older adults to dexterously manipulate fixed, low-friction surfaces in particular. 21 young and 18 older (65+ yrs) adults produced maximal voluntary contractions (MVCs) and steady submaximal forces (2.5 and 10% MVC) with the fingertip of the index finger. A Teflon covered custom-molded splint was placed on the fingertip. A three-axis force sensor was covered with either Teflon or sandpaper to create low- and high-friction surfaces, respectively. Maximal downward forces (F_z_) were similar (*p* = .135) for young and older adults, and decreased by 15% (*p*<.001) while pressing on Teflon compared to sandpaper. Fluctuations in F_z_ during the submaximal force-matching tasks were 2.45× greater (*p*<.001) for older adults than in young adults, and reached a maximum when older adults pressed against the Teflon surface while receiving visual feedback. These age-associated changes in motor performance are explained, in part, by altered muscle activity from three hand muscles and out-of-plane forces. Quantifying the ability to produce steady fingertip forces against low-friction surfaces may be a better indicator of impairment and disability than the current practice of evaluating maximal forces with pinch meters. These age-associated impairments in dexterity while interacting with low-friction surfaces may limit the use of the current generation of computing interfaces by older adults.

## Introduction

Mobile computing devices (e.g., smartphones) require dexterous manipulation against low-friction surfaces. Although it is known that, in general, manual dexterity is impaired in healthy older adults, which negatively influences their quality of life and ability to live independently [1], little is known about the ability of older adults to interact with fixed low-friction surfaces in particular. The majority of studies evaluating the influence of friction on hand motor control in older adults examine slip-grip responses and safety margins in fingertip force magnitudes generated in response to external perturbations of the object or the grasping of objects of varying frictional properties (e.g., [2], [3]). Although age-associated impairments have been identified using those experimental paradigms, far fewer studies have addressed the issue of dexterous manipulation against fixed, low-friction surfaces [4], [5], [6]. Furthermore, these studies are limited to examining maximal force production in young adults and report equivocal results. For example, maximal pinch force against low- vs. high-friction surfaces has both been reported to decrease [4] and stay the same [5]. In addition, the simultaneous production of fingertip motion and force against low-friction surfaces is particularly challenging for the central nervous system as force production may be limited due to neural factors independent of the biomechanical properties of muscle [6]. Thus, it is unclear how motor performance during maximal and submaximal tasks is influenced while pressing against fixed, low-friction surfaces, especially in older adults.

Impaired manual dexterity in older adults likely indicates problems in regulating fingertip force vector magnitude and direction. Specifically, there is evidence that producing well-directed forces is problematic for older adults [7], [8], [9]. In addition, fluctuations in fingertip force magnitude during isometric force-matching tasks are frequently greater in older than young adults, with performance depending on the task performed [10], [11], the type of visual feedback provided [12], [13], and the muscles/joints used [13]. Importantly, the functional relevance of examining force fluctuations to evaluate hand function has been established in older adults [10], [14]. Nonetheless, previous studies have not involved pressing against low-friction surfaces requiring well-directed forces to prevent slipping. As these low-friction interfaces are used in numerous devices (e.g., mobile devices, trackpads, and touchscreens), it is critical to assess potential age-associated impairments in manipulating low-friction surfaces.

The purpose of this study, therefore, was to examine whether motor performance during maximal and submaximal force pressing tasks in older adults was impaired relative to young adults while pressing against a low- vs. high-friction surface. If pressing against slippery surfaces is problematic for older adults, presumably due to the need to produce precise force magnitudes and directions to prevent slipping, it is expected that motor performance would show greater impairments in older than young adults when pressing against low- vs. high-friction surfaces. Young and older adults pressed against low- and high-friction surfaces to investigate age-related changes in: 1) MVC force magnitude and direction, 2) submaximal force fluctuations in normal and tangential forces with and without visual feedback, and 3) electromyogram (EMG) activity from three hand muscles during the MVC and submaximal force-matching tasks.

## Materials and Methods

### Ethics statement

The experiments were approved by the Institutional Review Board at the University of Wisconsin-Milwaukee. The local ethics committee approved consent for subjects ranging in age from 18–40 years and 65–90 years. All participants gave their written formal consent before participating in the study.

### Subjects

21 young (age: 21.4±3.7 years; range, 19–33 years; 10 females) and 18 older (age: 72.3±7.0 years; range, 65–87 years; 9 females), right-handed adults with no reported neuromuscular disorders or hand pathologies volunteered for the study.

### Experimental arrangement and procedures

**Figure 1:**
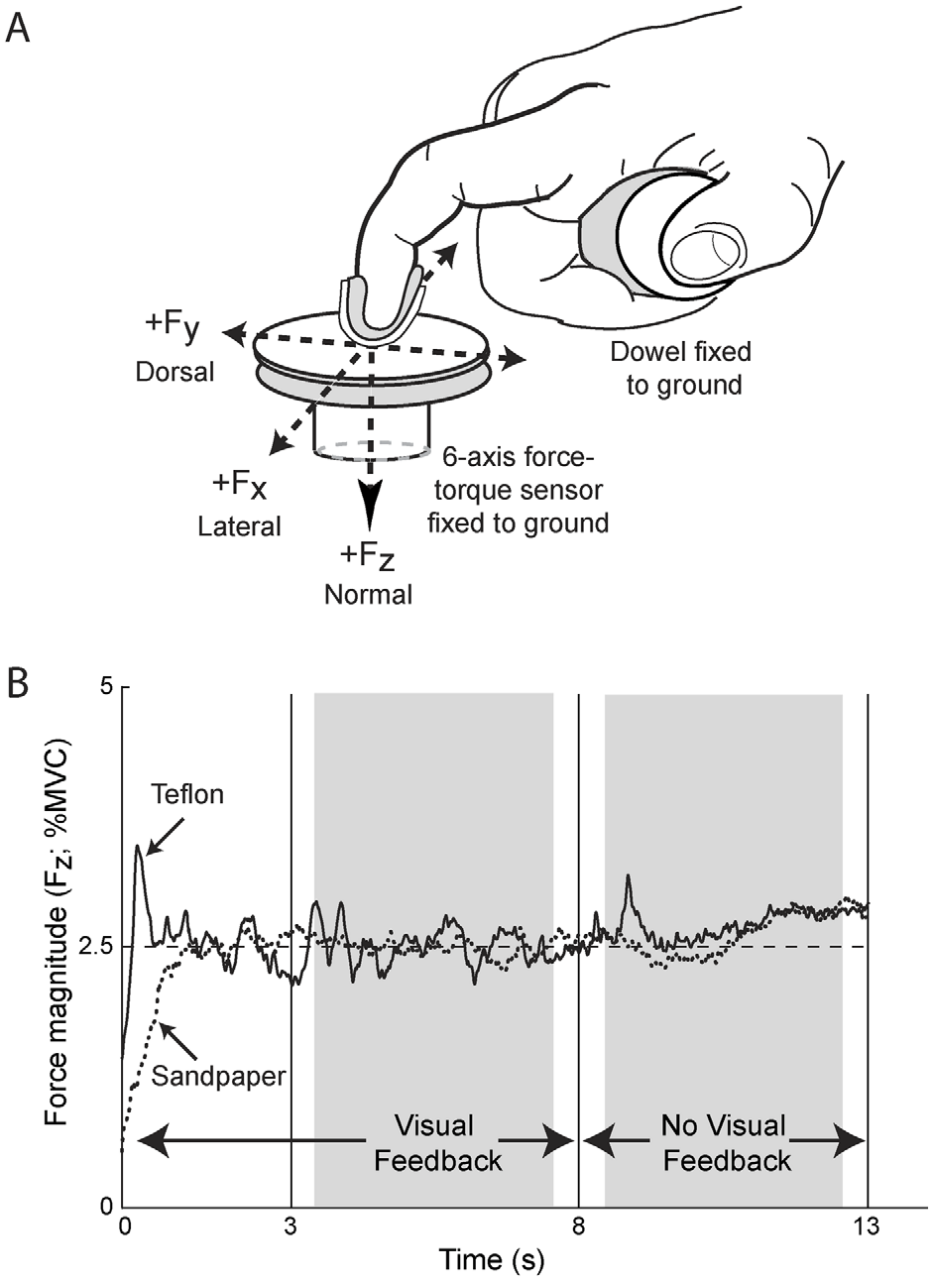
Hand setup and submaximal force-matching task. (*A*) Subjects pressed with a custom-molded thermoplastic splint on the index finger onto a force sensor; both surfaces were covered with Teflon or 320-grit sandpaper to create low- and high-friction conditions, respectively. Forces were measured normal to the sensor surface (F_z_), as well as in medial/lateral (F_x_) and dorsal/palmar (F_y_) directions. (*B*) Subjects increased force up to the target force (dashed line) within 3 s and held their force magnitude (black lines) as close to the target line as possible for 10 s. Gray highlighted areas indicate the time epochs where subjects received visual and no visual feedback.

Subjects were seated with their right forearm strapped to a horizontal platform (69 cm high) with a vacuum foam pad (Versaform pillow, Tumble Forms, Dolgeville, NY) to immobilize the elbow and forearm. As in Keenan et al. [6], subjects grasped a horizontal dowel with all fingers and thumb, except for the index finger, which was free to press on a three-axis force sensor (Nano 17, ATI Industrial Automation, Apex, NC; Figure 1). Forces were sampled at 1000 Hz (Spike2; Cambridge Electronic Design, UK). A low-friction Teflon surface was attached to a round pedestal (2.5 cm diameter) mounted on the force sensor. The sensor plane was oriented horizontally with downward normal forces denoted F_z_, and with the transducer rotated so that the x- and y-axes were in the medial/lateral (F_x_) and palmar/dorsal (F_y_) directions, respectively (lateral, dorsal, and downward forces corresponded to +N values; Figure 1). Sensor surface position and height were adjusted such that the index finger was in a neutral ad-abduction posture, the distal phalanx of the index finger was perpendicular to the sensor surface, and the proximal phalanx of the index finger was parallel to the sensor surface. As in our previous work to create a low-friction interface between the finger and sensor [6], subjects wore a custom-molded cover (i.e., thermoplastic material with rubber mesh insert for comfort) on the fingertip, with a thin Teflon strip secured along the centerline of the fingertip (Figure 1). The high-friction condition involved covering the Teflon surfaces with 320-grit sandpaper. The custom-molded thermoplastic splint: 1) enforced the fingertip force vector to remain oriented close to the normal force (F_z_) during the low-friction condition and provided minimal resistance to sliding (Teflon–Teflon friction coefficient  = 0.04), 2) helped remove the discontinuity at the fingernail, 3) reduced the possibility of pain during the MVCs, and 4) ensured that cutaneous feedback was similar across friction conditions. Visual feedback of force was provided on a 24-in LCD monitor located 1 m away and subjects viewed only F_z_ during all tasks. The order of the MVCs and submaximal force tasks for the low- and high-friction conditions was randomized across participants.

#### MVC task

Subjects were instructed to produce the largest F_z_ possible in both the low-friction and high-friction conditions. If peak F_z_ from two subsequent trials deviated by more than 5%, additional trials were performed. Subjects were asked after every trial if they believed their performance was maximal and subjects received strong verbal encouragement during the MVCs. The peak in F_z_ from the single highest trial was used to calculate the submaximal force levels.

#### Submaximal force-matching tasks

Subjects produced forces at 2.5% and 10% of peak F_z_ for both friction conditions. Visual feedback of force consisted of a line moving left to right across the screen in time and vertically based on the force produced by the subject (Figure 1B). A dashed black target line was positioned horizontally in the middle of the video monitor and subjects were instructed to keep their force magnitude as close as possible to the black target line. Each subject reported having normal or corrected to normal vision and to be able to clearly see the target line and force traces. Subjects were provided at least two practice trials for each condition. Each trial was 13 s in duration, and subjects completed two trials for each force level and friction condition. In each trial, subjects increased force from rest to the target force level within 3 seconds, and then held the force level for an additional 10 s (Figure 1B). Because vision has been shown to preferentially increase force fluctuations in older adults relative to young adults performing force-matching tasks [13], we removed visual feedback of force after 8 s (Figure 1B).

#### Muscle activity

A 16-channel linear EMG array (EMG-USB2; OT Bioelettronica, Torino, Italy) was used to place surface EMG electrodes (see [11] for experimental details) on the skin overlying *first dorsal interosseous* (FDI), *extensor digitorum communis* (EDC), and *flexor digitorum superficialis* (FDS). Briefly, bipolar electrode pairs (4-mm diameter, silver-silver chloride; 15 mm inter-electrode distance) were positioned on the skin 15 and 30 mm distal to the estimated location of the innervation zone and in line with muscle fiber direction. EMG signals were amplified (1K; Coulbourn Instruments, Whitehall, PA) and band-pass filtered (13–1 KHz) using an isolated bio-amplifier. To normalize EMG data during the force pressing tasks, subjects performed brief 3–5 s maximal contractions of the three hand muscles while the experimenter provided manual resistance to the index finger at the start and end of the experiment (see [11]).

### Data analysis

For the MVC tasks, the primary dependent variable was the peak F_z_ produced during MVC trials against the low- and high-friction surfaces. Peak F_z_ values from the two MVC trials were averaged and compared across friction conditions and subject ages. In addition, corresponding values of F_x_ and F_y,_ when F_z_ was maximal were calculated and compared across conditions.

For the submaximal forces, the coefficient of variation in F_z_ (CV  =  SD of F_z_/mean F_z_ ×100) was calculated from 3.5–7.5 s during the epoch of visual feedback, and from 8.5–12.5 s during the epoch of no visual feedback. As commonly done (e.g., [12], [13]), force output during those two epochs was detrended by removing the linear trend from the force data, as drift during the no visual feedback condition could influence force variability (e.g., see Fig. 1B). CV values from the two trials were averaged and compared across age groups, friction conditions, and visual feedback conditions. The standard deviation of F_x_ and F_y_ for both epochs was also calculated and compared across conditions.

Additionally, normalized average full-wave rectified EMG amplitudes were calculated for all tasks. For MVC tasks, EMG amplitudes were calculated for 500 ms centered on the peak in F_z_. For the submaximal steadiness tasks, EMG amplitudes were calculated for the 4 s epoch of vision and no vision.

### Statistical analysis

For MVC tasks, six mixed between-within subjects ANOVAs were conducted separately for F_z_, F_x_, and F_y_, and EMG amplitudes (FDI, EDC, and FDS) with repeated measures on friction condition, and with the between-subjects factor of age group. For submaximal force steadiness tasks, six mixed between-within subjects ANOVAs were conducted for the CV in F_z_, the standard deviation of F_x_ and F_y_, and EMG amplitudes (FDI, EDC, and FDS) with repeated measures on friction condition, force level, and visual feedback, and with between-subjects factor of age group. Alpha level for all statistical tests was *p*<.05. Significant interactions were followed by post-hoc analyses (t-test with Bonferroni corrections). Results are presented as mean ± SD in the text and standard error (SE) in the figures.

## Results

### MVC tasks

**Figure 2:**
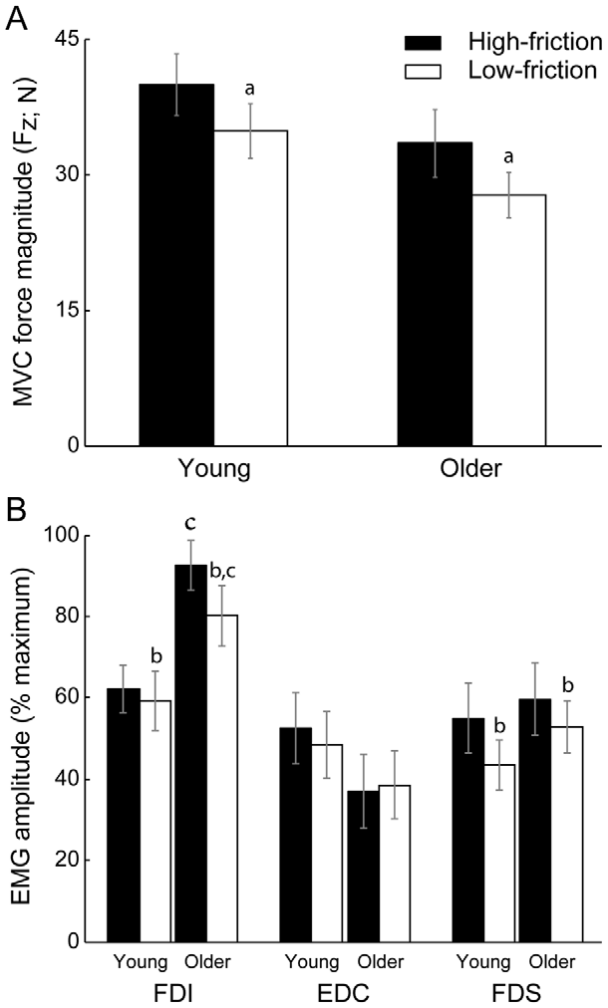
Summary data for maximal voluntary contraction (MVC) forces and electromyogram (EMG) activity. (*A*) Maximal downward forces (F_z_) decreased similarly for young and older adults when pressing on a low- vs. high-friction surface. (*B*) Along with the decrease in F_z_ across friction conditions, average full-wave rectified EMG from *first dorsal interosseous* (FDI) and *flexor digitorum superficialis* (FDS) decreased while pressing on the high vs. low-friction surface, and FDI EMG was increased in older vs. young adults. There was no change in EMG activity in *extensor digitorum communis* (EDC) across conditions. Values are means ± SE. ^a^ *p*<.001 vs. high-friction; ^b^ *p*<.05 vs. high-friction; ^c^ *p* = .006 vs. young adults.

Peak F_z_ magnitudes decreased (*F*_1,37_ = 19.55; *p*<.001) by 15.0% for the low-friction Teflon condition compared with the high-friction sandpaper condition (31.22±13.29 N and 36.71±14.88 N, respectively; Figure 2A). There was no significant difference (*F*_1,37_ = 2.34; *p* = .135) across young and older adults (37.38±18.47 N and 30.55±19.95 N, respectively; Figure 2A) or on interaction between age group and friction condition (*F*_1,37_ = 0.05; *p* = .833). For tangential forces during MVCs, although there was no change (*F*_1,37_ = 3.87; *p* = .057) in F_x_ between low- and high-friction conditions (0.98±5.97 N and 0.28±1.73 N, respectively), F_y_ was greater (*F*_1,37_ = 6.80; *p* = .013) and directed dorsally when producing MVC forces against the sandpaper surface (1.47±3.24 N) relative to the Teflon surface (0.09±1.46 N). There were no age group main effects (*p*>.236) or significant interactions (*p*>.201) for F_x_ and F_y_.

EMG amplitude in FDI decreased (*F*_1,37_ = 4.58; *p* = .039) during MVCs against Teflon relative to sandpaper, and increased (*F*_1,37_ = 8.592; *p* = .006) for older compared with young adults (Figure 2B); there was no significant interaction between age group and friction condition (*F*_1,37_ = 1.68; *p* = .203). In addition, the only other significant change in muscle activity during MVCs was that EMG amplitude in FDS decreased (*F*_1,37_ = 6.15; *p* = .018) during MVCs against Teflon relative to sandpaper (Figure 2B).

### Submaximal force-matching tasks

**Figure 3:**
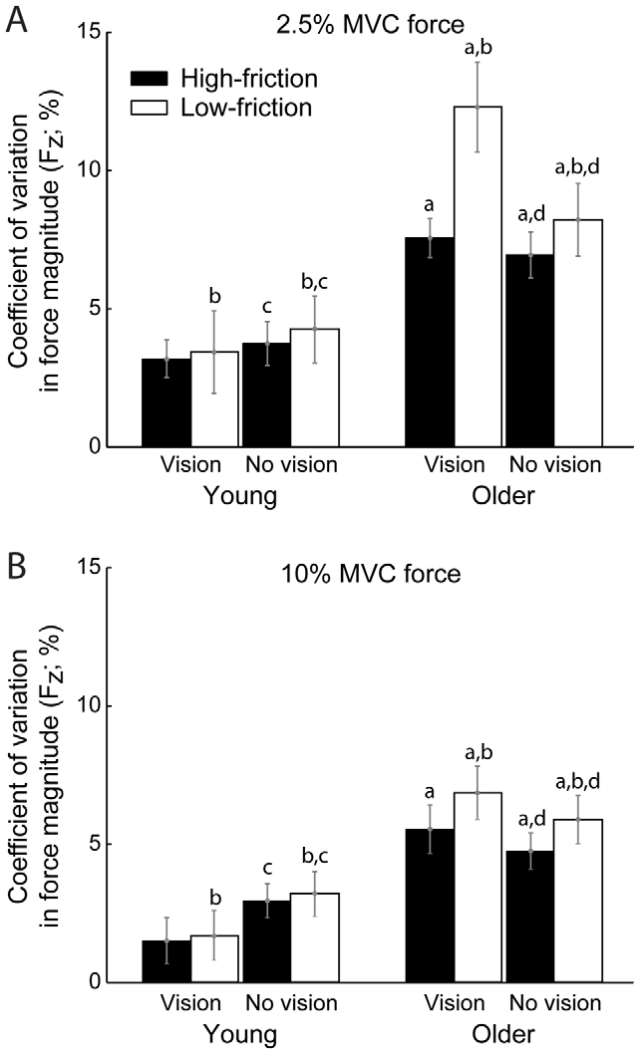
Summary data for fluctuations in force during submaximal force-matching tasks. Fluctuations in downward forces (coefficient of variation in F_z_) during submaximal force tasks were increased in older relative to young adults for both 2.5% (*A*) and 10% peak F_z_ (*B*) force-matching tasks, and while pressing against a low-friction relative to a high-friction surface. There was also an age by vision interaction (*p* = .002), with greater force fluctuations in older adults when vision was present, and lesser force fluctuations in young adults when vision was present. Values are means ± SE. ^a^ *p*<.001 vs. young adults; ^b^ *p* = .023 vs. high-friction. ^c^ *p* = .05 vs. vision in young adults; ^d^ *p* = .008 vs. vision in older adults.

Figure 3 shows that the CV of force (F_z_): 1) increased (*F*_1,37_ = 23.06; *p* = .001) in older compared with young adults, 2) increased (*F*_1,37_ = 5.59; *p* = .023) while pressing against Teflon relative to sandpaper, and 3) increased (*F*_1,37_ = 14.9; *p* = .001) while pressing with 2.5% vs. 10% peak F_z_. There was also a significant age group by vision interaction (*F*_1,37_ = 11.69; *p* = .002), with the CV of F_z_ for older adults increased (*p* = .008) for the vision compared with no vision condition, and the CV of F_z_ for young adults decreased (*p* = .05) during the vision compared with no vision condition. There were no other significant interactions (*p*>.081) for the CV of F_z_. Thus, fluctuations in F_z_ were greatest when older subjects pressed against the low-friction surface at 2.5% peak F_z_ with visual feedback (Figure 3).

Related to force fluctuations in tangential forces during submaximal tasks, the SD of force magnitude in F_x_ was increased (*F*_1,37_ = 5.32; *p* = .027) in older (0.036±0.025 N) relative to young subjects (0.023±0.023 N) and also increased (*F*_1,37_ = 38.88; *p* = .001) at the 10% peak F_z_ (0.043±0.03 N) compared with the 2.5% peak F_z_ level (0.015±0.008 N). However, the increase in SD of force with age in F_x_ was qualified by an age group by vision interaction (*F*_1,37_ = 5.26; *p* = .028). Specifically, the SD of F_x_ was increased (*p* = .008) in older relative to young adults when visual feedback was provided (0.037±0.026 N and 0.021±0.025 N, respectively), but not significantly different (*p* = .108; 0.034±0.026 N and 0.024±0.024 N, respectively) without visual feedback. There were no significant changes (*p*>.189) in F_y_ during the submaximal force tasks. Thus, force fluctuations in F_x_ and F_y_ were not significantly different across the low- and high-friction conditions.

**Figure 4:**
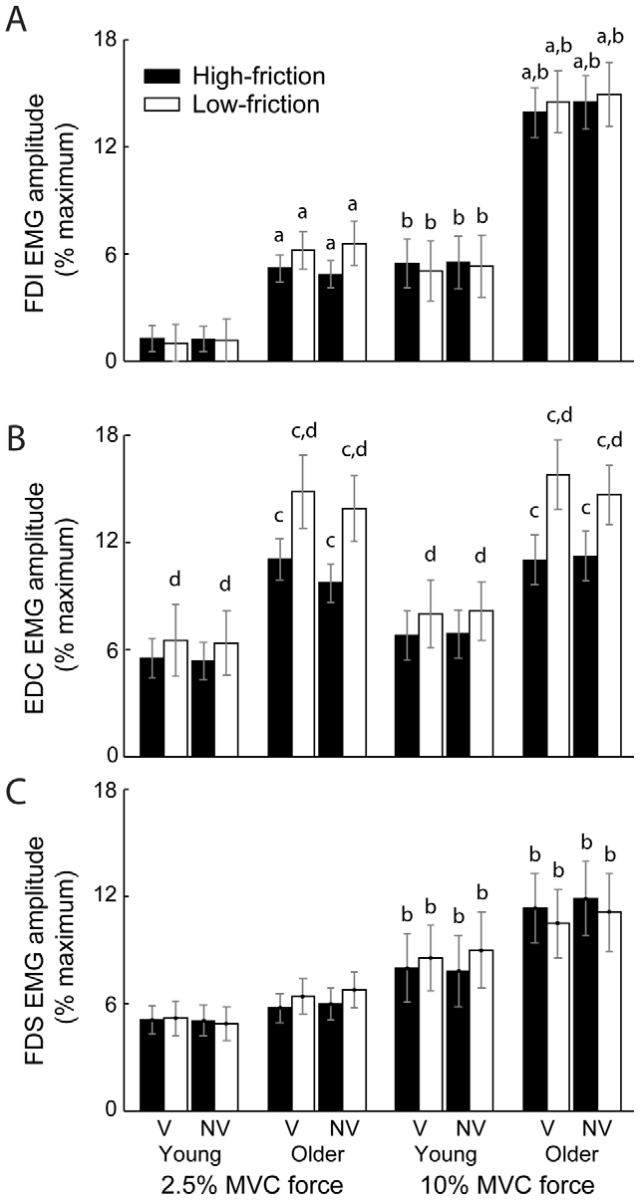
Summary data for electromyogram (EMG) activity during submaximal force-matching tasks. Older adults had greater EMG activity than young adults for *first dorsal interosseous* (FDI) (*A*) and *extensor digitorum communis* (EDC) (*B*) while performing submaximal steadiness tasks. EMG activity also increased in EDC when pressing against a low-friction vs. high-friction surface, potentially to stabilize the fingertip while pressing against the slippery Teflon surface. In contrast to EDC, EMG amplitude also increased in FDI and *flexor digitorum superficialis* (FDS) while pressing at the 10% vs. 2.5% MVC force level. Thus, altered submaximal force fluctuations were accompanied by changes in muscle activation strategies by young and older adults. Values are means ± SE. ^a^ *p*<.001 vs. young adults; ^b^ *p*<.001 vs. 2.5 MVC force; ^c^ *p* = .002 vs. young adults; ^d^ *p* = .006 vs. high-friction surface.

EMG amplitude in FDI (Figure 4A) during the submaximal tasks was: 1) increased (*F*_1,37_ = 18.39; *p* = .001) in older compared to younger adults, 2) increased (*F*_1,37_ = 78.31; *p*<.001) while pressing at 10% vs. 2.5% peak F_z_, and 3) similar (*F*_1,37_ = 0.5; *p* = .484) for sandpaper and Teflon surfaces. EMG amplitude in EDC (Figure 4B) was increased (*F*_1,37_ = 10.63; *p* = .002) in older compared to young adults and increased (*F*_1,37_ = 8.59; *p* = .006) while pressing against Teflon relative to sandpaper. EMG amplitude in FDS (Figure 4C) was increased (*F*_1,37_ = 10.63; *p*<.001) while pressing at 10% vs. 2.5% peak F_z_, with a significant (*F*_1,37_ = 4.53; *p* = .04) interaction between force level and vision condition (Figure 4C). All other main effects and interactions were not significant (*p*>.07).

## Discussion

The key findings of the present study are as follows. First, submaximal force fluctuations were increased in older adults relative to young adults, especially for the condition that involved pressing against a fixed, low-friction surface with visual feedback at low force magnitudes. Second, impaired cutaneous sensation was not the primary factor influencing impaired motor performance in the older adults while pressing against the low- vs. high-friction surface as subjects wore a thermoplastic splint to ensure that cutaneous feedback was similar across the two friction conditions. Third, peak F_z_ magnitudes decreased similarly for young and older adults while pressing against low- vs. high-friction surfaces. Fourth, EMG activation patterns across FDI, EDC, and FDS were altered while pressing against the low- vs. high-friction surface. Taken together, these findings are consistent with the concept that interacting with low-friction surfaces is challenging, especially for older adults. Based on performance on MVC and submaximal tasks, quantifying the ability to produce submaximal finger-tip forces against low-friction surfaces may be a better indicator of impairment and disability than the common practice of evaluating stable forces with pinch grip meters. These results also likely indicate potential difficulties that older adults may encounter when trying to use computing interfaces that employ low-friction surfaces.

### Steadiness of submaximal forces

The functional relevance of force fluctuations has been previously established in older adults [10], [14]. In the current study, fluctuations in force magnitude were maximal when older adults pressed at the 2.5% peak F_z_ level with visual feedback against the Teflon surface (Figure 3). Indeed, CV of force for this condition was 12.3±9.9%, which is more similar to that reported for stroke patients during fatiguing contractions [15] than healthy older adults where values typically range from 4–8% at low force levels [10], [11]. Although the CV of force is generally greatest at low forces [11], [13], especially when visual feedback is provided [13], force variability may have further increased in older adults pressing against Teflon due to: 1) an inability to stabilize the position of the finger and control variability in tangential directions to the normal force to avoid slipping and 2) impaired sensation. These two possibilities are discussed below.

First, a number of approaches consider variability as efficient motor control (e.g., ‘uncontrolled manifold’ [16] and ‘minimum intervention’ [17] hypotheses). In these approaches, the sensorimotor system preferentially controls task-relevant parameters while allowing task-irrelevant parameters to fluctuate. In the current study, variability in F_z_ is explicitly the task-relevant parameter, while the task relevance of variability in F_x_ and F_y_ depends on friction condition. Specifically, fine control of tangential forces is critical when pressing against a low-friction surface to prevent slipping [17], though not as important when pressing against sandpaper. Thus, by changing the friction condition the nature of the task and the task-relevant parameters are also changed, placing greater demands on the central nervous system and potentially resulting in concomitant increases in the CV of normal forces as the task becomes more challenging. Consistent with this interpretation, Shinohara et al. [18] analyzed the covariation of force within the ‘uncontrolled manifold’ and found that young adults better stabilized force than older adults when pressing with the fingers against a stable manipulandum. In addition, the submaximal tasks performed in the current study were certainly challenging for older adults. Specifically, in older relative to young adults force fluctuations in the dorsal/palmar direction increased by 56.5% and EMG activity in FDI and EDC increased by 310% and 191%, respectively. Interestingly, there were no significant differences in tangential force fluctuations between sandpaper and Teflon surfaces in the current study, though EDC EMG activity increased when pressing against Teflon relative to sandpaper surfaces, potentially to help stabilize the finger against the slippery Teflon surface. Nonetheless, pressing against sandpaper and Teflon surfaces may not be different enough to highlight the difference in tangential force fluctuations. One possibility that could address this limitation in future work is to rigidly fix the fingertip to the target surface, thereby making the control of tangential forces unnecessary, in contrast to the sandpaper condition used in the current study.

Second, in addition to vision, other sensory modalities (e.g., cutaneous sensation and proprioception) are impaired in older adults (e.g., [2], [3]) and may influence steady force production against low-friction surfaces. For example, cutaneous sensation is impaired in older adults and influences slip-grip responses [3]. Partly for this reason, we used a thermoplastic finger splint so that cutaneous sensation did not vary across friction conditions. However, by minimizing cutaneous sensation with the finger splint, other impaired sensory modalities (e.g., proprioception and vision) could be more heavily relied upon and similarly impair performance. In addition, visual feedback in the current study was of fingertip force magnitude, not visual feedback of motion as is frequently employed during activities of daily living. Given the differences between our experimental approach and more ecological tasks, further mechanistic studies are needed to examine the role of impaired sensation in older adults to influence manipulation of low-friction surfaces.

### MVC forces

Peak F_z_ forces were statistically similar (*p* = .135) in young and older adults and the ∼15.0% decrease (*p*<.001) in peak F_z_ force while pressing against Teflon compared with sandpaper surfaces was similar across age groups (Figure 2A). Similarly, Seo et al. [4] found a 10% decrease in pinch grip force in young subjects pressing against a low-friction paper surface compared to a high-friction rubber surface. Interestingly, the decrease in peak F_z_ across friction surfaces in the current study was independent of subject age, suggesting that the low-friction surface was not more problematic for older relative to young adults, at least while producing maximal forces. Also, the 15.0% decrease in peak F_z_ was accompanied by a 9.8% and 16.1% decline in FDI and FDS EMG amplitudes (Figure 2B), respectively. Therefore, the reduction in peak F_z_ across friction conditions may be a voluntary response to limit forces against the slippery Teflon surface. Alternatively, fingertip forces were directed dorsally relative to the normal force by 2.1° vs. 0.13° while pressing against sandpaper and Teflon, respectively. As discussed in Valero-Cuevas et al. [19], the change in fingertip force direction across friction conditions could have resulted in a change in the activation pattern across the seven muscles of the index finger to maximize force. Nonetheless, the current study was limited to examine EMG from only three muscles. Lastly, FDI EMG amplitude increased (*p* = .006) by 42.3% in older compared to young adults (Figure 2B). As older adults may experience a preferential weakening of intrinsic vs. extrinsic hand muscles [8], [20], this increased activity in FDI, an intrinsic hand muscle, could be a compensatory response to account for the decreased muscle strength of the intrinsic hand muscles.

### Conclusion

Maximum pinch grip is commonly evaluated clinically in older adults, though it may not be the most sensitive measure available to assess hand function. For example, in the current study older subjects produced similar (*p* = .135) maximal force magnitudes as young adults, but submaximal force fluctuations were 3.7× greater in older adults relative to young adults pressing against the low-friction surface with visual feedback. Thus, motor tasks that demand precision in directing fingertip forces may be a more sensitive metric to assess motor function in older adults. For example, the Strength-Dexterity test assesses the capacity to accurately direct pinch forces by pressing and compressing different springs [7], and older adults had impairments relative to young adults in their ability to accurately direct forces. The current study extends that result and finds that motor performance is also impaired when well-directed forces on fixed, low-friction surfaces are required. Also, although we did not directly assess the ability of older adults to manipulate low-friction computing interfaces, the results of this study may be pertinent. Specifically, interacting with the low-friction surfaces in these devices requires older adults to produce steady, accurate, and well-directed forces at low forces with visual feedback guiding performance, a similar set of conditions that older adults struggled with in the current study. Thus, future work should explore the potential limitations older adults may have interacting with mobile computing devices and develop ergonomic aids and training interventions to improve performance.
